# Evaluation of Clinical Outcomes and Recurrence After Surgical Excision of Oral Leukoplakia: A Prospective Cohort Study

**DOI:** 10.7759/cureus.71593

**Published:** 2024-10-16

**Authors:** Lakhan Talreja, Rohit Goyal, Divya Yadav, Navneet Singh, Sangita Kalita, Sneha B Jaiswal

**Affiliations:** 1 Department of Oral and Maxillofacial Surgery, Maharaja Ganga Singh Dental College and Research Centre, Sri Ganganagar, IND; 2 Department of Prosthodontics, Maharaja Ganga Singh Dental College and Research Centre, Sri Ganganagar, IND

**Keywords:** excision, leukoplakia, recurrence, surgical, tobacco

## Abstract

Objective: This study aimed to evaluate the clinical outcomes and recurrence patterns in cases of surgical excision of oral leukoplakia and identify key factors associated with disease recurrence and malignant transformation.

Materials and methods: Thirty patients aged 18-70 years who were diagnosed with oral leukoplakia through clinical and histopathological evaluation, with lesions larger than 1 cm^2^ requiring surgical excision, and who had ceased tobacco use or irritant habits for at least two months before surgery were considered. All the patients were monitored for 18 months postoperatively. Recurrence was defined as the reappearance of leukoplakia at or near the surgical site. Postoperative complications, including infection, scarring, and functional impairments affecting speech or mastication, were comprehensively documented at each follow-up: one week, one month, three months, six months, 12 months, and 18 months after surgery. The data were subjected to statistical analysis.

Results: Overall, 27% of patients experienced recurrence, with higher recurrence rates in nonhomogenous lesions (40%), tobacco users (35%), and dysplastic lesions (100%). Factors such as a history of tobacco use and histopathological dysplasia were strongly associated with an increased risk of recurrence.

Conclusion: This study highlighted the significance of lesion type, dysplasia, and patient risk factors such as tobacco use in predicting postsurgical recurrence. Close follow-up and risk factor modification are recommended to optimize patient outcomes.

## Introduction

Oral leukoplakia is the most common potentially malignant disorder of the oral cavity and is characterized by white patches or plaques that cannot be clinically or pathologically defined as any other condition [1]. The World Health Organization defines oral leukoplakia as "a white plaque of questionable risk having excluded (other) known diseases or disorders that carry no increased risk for cancer" [2]. Clinically, it can be classified as homogeneous leukoplakia, presenting as flat, thin, white patches, and nonhomogeneous leukoplakia, which includes speckled, nodular, and verrucous forms that are more likely to undergo malignant transformation [3].

The global prevalence of oral leukoplakia ranges from 1% to 5%, with regional variation [4]. In India, it is particularly common due to high tobacco consumption, with prevalence rates ranging from 0.2% to 4.9% [5]. The incidence is higher among males, reflecting the demographic pattern of tobacco use [6]. In addition to tobacco use, other causative factors include alcohol consumption, chronic irritation from dental prostheses, betel quid chewing, and poor oral hygiene. Human papillomavirus infection has also been implicated in some cases, although its role is less well defined than other mucosal disorders [6].

Treatment modalities for oral leukoplakia range from conservative management, such as cessation of causative habits, to medical and surgical interventions. Conservative approaches include vitamin A derivatives, such as retinoids and antioxidants, and antifungal therapy in cases of Candida superinfection [7]. However, these conservative treatments often have limited efficacy in preventing malignant transformations. The mainstay of treatment for high-risk leukoplakia is surgical excision, which can be performed using various techniques, including conventional scalpel surgery, laser ablation, and cryotherapy [8]. Each method has advantages and limitations. Scalpel excision allows for histopathological examination of the lesion but carries risks of infection, scarring, and recurrence [9,10]. Laser ablation offers a bloodless field and faster healing but is associated with higher recurrence rates in some cases [10]. Cryotherapy is less invasive but may not be suitable for larger lesions or those with significant dysplasia [11].

Despite the availability of these treatments, oral leukoplakia poses a therapeutic challenge because of its unpredictable nature and risk of recurrence. Recurrence rates following surgical excision vary widely, ranging from 10% to 35%, depending on the lesion type, size, and excision technique [12,13]. Furthermore, oral leukoplakia has significant malignant potential, with studies reporting malignant transformation rates between 1% and 20% [14]. Nonhomogeneous and dysplastic leukoplakia are particularly prone to malignant changes, underscoring the importance of early and adequate treatment [8].

Surgical excision is indicated for lesions that exhibit dysplasia and nonhomogeneous appearance, or fail to regress after eliminating causative factors [15]. While excision can reduce the risk of malignant transformation, it does not completely eliminate this risk, particularly in cases of high-grade dysplasia [16]. The multifactorial etiology and progressive nature of the disease necessitate long-term follow-ups. Monitoring patients for recurrence or malignant transformation is critical, especially in the first few years after surgery, when the risk is the highest [14,16]. Regular follow-up allows for early detection of recurrent or new lesions, potentially improving patient outcomes by facilitating prompt intervention. This study aimed to evaluate the clinical outcomes of surgical excision for oral leukoplakia over an 18-month follow-up period. Extended follow-up is essential for determining the recurrence rate, assessing the effectiveness of surgical treatment, and monitoring malignant transformation.

## Materials and methods

Study design and setting

This study was conducted at the Department of Oral Surgery at Maharaja Ganga Singh Dental College and Research Centre, Sri Ganganagar, from March 2022 to February 2024. Institutional Ethical Committee approval was obtained (MGSDC/SY/22/012), and written informed consent was obtained from all participants before inclusion in the study. This study adhered to the ethical principles outlined in the Declaration of Helsinki, ensuring the protection of the participant's rights, safety, and well-being throughout the research. All procedures were conducted strictly with these ethical guidelines, emphasizing the importance of patient autonomy, confidentiality, and informed participation in clinical research. The patients were observed after surgical excision and cessation of habits for treatment of oral leukoplakia. The researchers did not perform any intervention.

Sample size estimation

The sample size for the study was estimated using Cramér's phi ratio of 1.89 to account for the recurrence potential of leukoplakia [13]. Based on this ratio, a sample size of 30 patients was determined to be sufficient to achieve 80% statistical power with a 5% alpha error, ensuring the reliable detection of significant differences. The calculations were performed using the G*Power software version 3.6.9 (Heinrich-Heine-Universität Düsseldorf, Düsseldorf, Germany).

Inclusion and exclusion criteria

Inclusion criteria included patients aged 18-70 years diagnosed with oral leukoplakia, confirmed via clinical and histopathological evaluations. Lesions larger than 1 cm^2^ treated with surgical excision were considered. Only patients who had ceased tobacco use or other causative habits for at least two months before surgery and who consented to an 18-month follow-up were included. The reason for this criterion was that the irritant habits can maintain a chronic inflammatory state in oral tissues, which may interfere with the body's ability to heal after surgery. Moreover, by stopping the habit, clinicians can monitor whether the leukoplakic lesion improves or regresses on its own. In some cases, cessation of the irritant might lead to partial or complete regression of the leukoplakia, reducing the need for surgical intervention. Exclusion criteria included patients with systemic conditions, such as diabetes mellitus or immunocompromised states that could impair healing, pregnant or lactating women, individuals with leukoplakia associated with other disorders, such as candidiasis or lichen planus, those who had undergone prior surgical treatment for leukoplakia, and patients unwilling or unable to comply with postoperative follow-up.

Methodology

**Figure 1:**
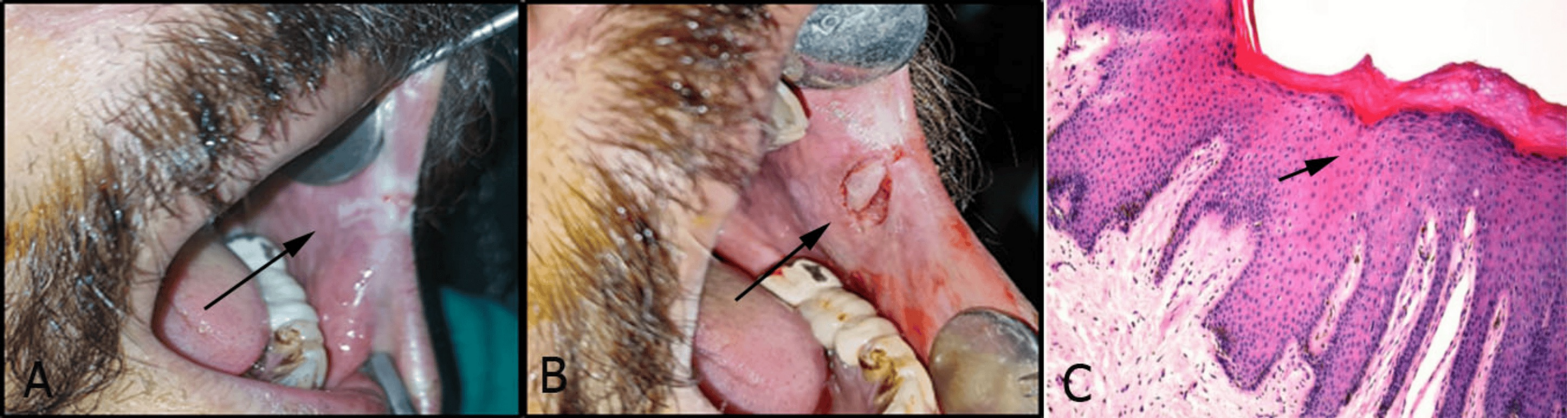
(A) Oral leukoplakia of buccal mucosa. (B) Surgical excision of the lesion. (C) Histopathology of oral leukoplakia

Preoperative assessment included a thorough clinical evaluation of the lesion, habit history, and relevant medical history by a single trained and calibrated researcher to achieve reliable, consistent, and clinically relevant preoperative assessments for patients with oral leukoplakia. The lesion size, type (homogeneous or nonhomogeneous), and clinical features were documented. A histopathological biopsy confirmed the diagnosis and graded dysplasia if present (Figure 1).

The counseling sessions were performed regularly with all patients who were diagnosed with oral leukoplakia, and only those who left their habit for at least two months before the surgical procedure were included. All patients underwent surgical excision of oral leukoplakia, along with continued counseling sessions for cessation of habits (alcohol consumption or tobacco use).

Thirty patients were selected based on the eligibility criteria who agreed to an 18-month follow-up. Postoperative assessment was conducted at multiple intervals to monitor healing, recurrence, and complications. The surgical site was evaluated clinically at one week, one month, three months, six months, 12 months, and 18 months after surgery. Wound healing was assessed based on signs of infection, dehiscence, and granulation tissue formation. Recurrence was defined as the reappearance of leukoplakia at or near the surgical site, assessed at each visit. Malignant transformation was evaluated via biopsy in cases of suspicious lesions. Postoperative complications, including infection, scarring, and functional impairments affecting speech or mastication, were comprehensively documented during each follow-up. This methodology ensured detailed monitoring of the clinical outcomes over an 18-month follow-up after surgical excision.

Statistical analysis

Descriptive statistics, including the mean and standard deviation, were calculated for continuous variables such as lesion size and pain scores. In contrast, categorical variables such as recurrence and complications were expressed as frequencies and percentages. Binomial tests were used to compare the recurrence and complication frequencies in the cohort. All analyses were performed using statistical software (IBM Corp. Released 2013. IBM SPSS Statistics for Windows, version 22.0., IBM Corp., Armonk, NY). Additionally, Kaplan-Meier survival analysis was conducted to evaluate the time to recurrence or malignant transformation over the 18-month follow-up period.

## Results

This study analyzed various variables using a binomial test to determine the distribution of each category. Sex distribution was significantly different, with 21 (70%) males being more prevalent than nine (30%) females (p = 0.043). Regarding smoking habits, 17 (57%) participants were smokers, and 13 (43%) were nonsmokers, without a significant difference. Twenty (67%) participants were tobacco users, and 17 (33%) were nontobacco users; however, the difference was not statistically significant (p = 0.099). The lesions were predominantly nonhomogenous in 20 (67%) patients and occurred more frequently in the lingual mucosa, particularly on the floor of the mouth. Infection and scarring were observed in fewer cases, with a significant difference (p = 0.001). Recurrence and dysplasia were observed in eight (27%) cases. Malignancy and new lesions were rare but significant (p = 0.001). These findings highlighted critical variables that could influence disease progression (Table 1).

**Table 1:** Analysis of various variables to determine the distribution of each category Data are presented as N (%) ^*^p <0.05: significant ^**^Binomial test was used for all categorical variables except sex, type of lesion, and site of lesion for which a chi-square test was used CI: confidence interval

Variables	Category	Frequency, N (%)	p value^**^	95% CI for proportion (lower limit to upper limit)
Sex	Female	9 (30%)	0.043^*^	0.147-0.494
	Male	21 (70%)		0.506-0.853
Smoking	No	13 (43%)	0.585	0.255-0.626
	Yes	17 (57%)		0.374-0.745
Tobacco use	No	10 (33%)	0.099	0.173-0.528
	Yes	20 (67%)		0.472-0.827
Alcohol use	No	18 (60%)	0.362	0.406-0.773
	Yes	12 (40%)		0.227-0.594
Type of lesion	Homogenous	10 (33%)	0.099	0.173-0.528
	Nonhomogenous	20 (67%)		0.472-0.827
Site of lesion	Buccal mucosa	12 (40%)	0.362	0.227-0.594
	Lingual mucosa	18 (60%)		0.460-0.773
Infection	No	24 (80%)	0.001^*^	0.614-0.923
	Yes	6 (20%)		0.077-0.386
Scarring	No	24 (80%)	0.001^*^	0.614-0.923
	Yes	6 (20%)		0.077-0.386
Recurrence	No	22 (73%)	0.016^*^	0.541-0.877
	Yes	8 (27%)		0.123-0.459
Development of new lesion	No	27 (90%)	0.001^*^	0.735-0.979
	Yes	3 (10%)		0.021-0.265
Dysplasia	No	22 (73%)	0.016^*^	0.541-0.877
	Yes	8 (27%)		0.123-0.459
Malignancy	No	25 (83%)	0.001^*^	0.653-0.944
	Yes	5 (17%)		0.056-0.347

**Figure 2:**
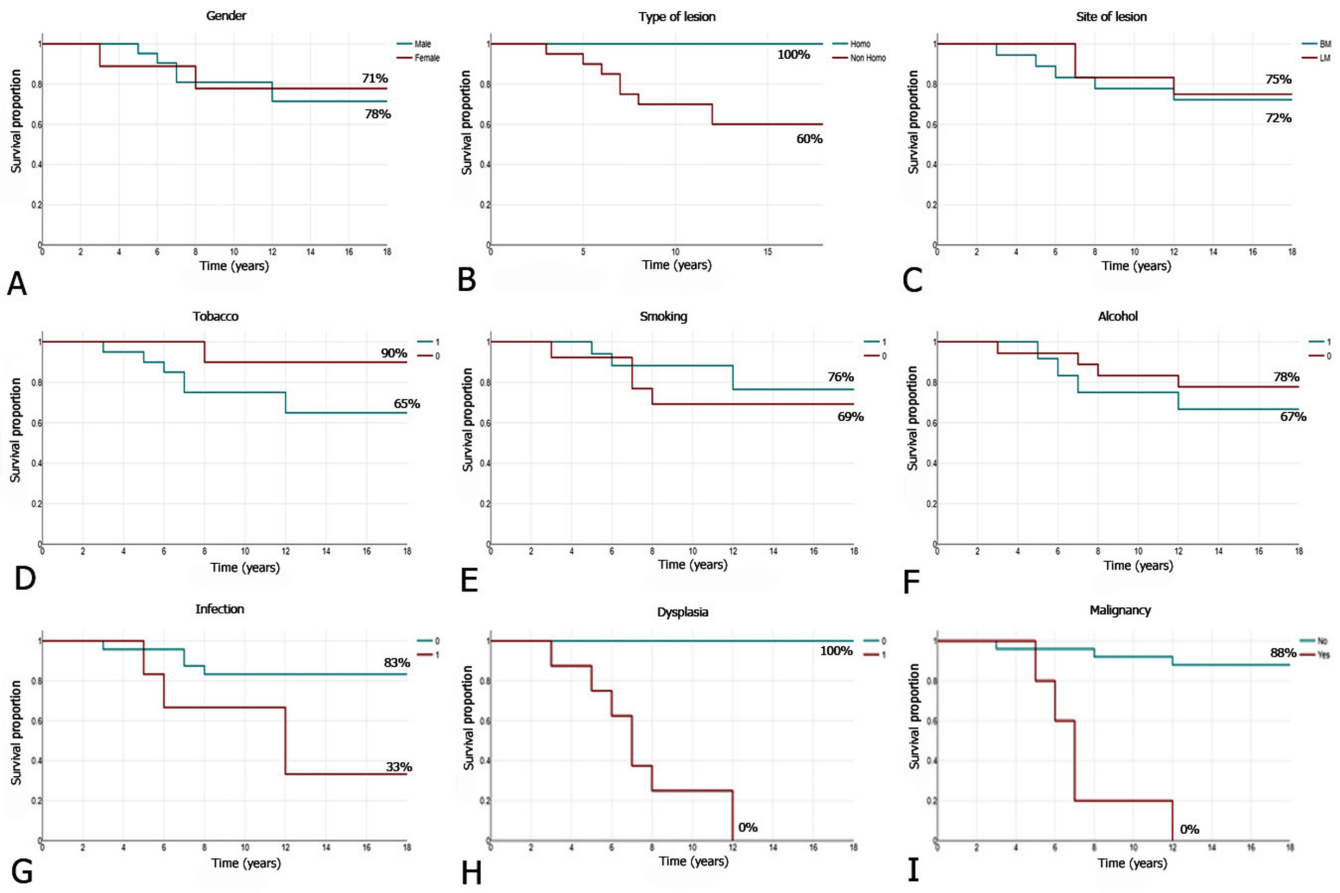
Kaplan-Meier analysis for survival of participant from recurrence considering different variables. (A) Sex, (B) type of lesion, (C) site of lesion, (D) tobacco, (E) smoking, (F) alcohol, (G) infection, (H) dysplasia, and (I) malignancy

Kaplan-Meier survival analysis revealed notable differences in leukoplakia recurrence across the various categories. Regarding sex, six (29%) males and two (22%) females experienced recurrence. Nonhomogenous lesions had eight (40%) events, whereas no recurrences were observed in homogenous lesions. Regarding the lesion site, recurrence occurred in five (28%) cases in the buccal mucosa and three (25%) in the labial mucosa. Seven (35%) tobacco users were associated with recurrence compared to only one (10%) nonuser. Smokers and nonsmokers showed four events each. Similarly, alcohol users and nonusers reported four (33%) and four (22%) cases of recurrence, respectively. Patients with infections showed a high recurrence rate. Dysplasia was linked to all eight (100%) recurrences in its category, while malignancy accounted for five (100%) events, highlighting significant disease progression in these groups (Table 2, Figure 2).

**Table 2:** Survival analysis without recurrence using Kaplan-Meier test Data are presented as N (%)

Variables	Category	Total, N (%)	Recurrence, N (%)	Nonrecurrence, N (%)
Sex	Male	21 (70%)	6 (29%)	15 (71%)
	Female	9 (30%)	2 (22%)	7 (78%)
Site of lesion	Homogenous	10 (33%)	0 (0%)	10 (100%)
	Nonhomogenous	20 (67%)	8 (40%)	12 (60%)
Type of lesion	Lingual mucosa	18 (60%)	5 (28%)	13 (72%)
	Buccal mucosa	12 (40%)	3 (25%)	9 (75%)
Tobacco	Yes	20 (67%)	7 (35%)	13 (65%)
	No	10 (33%)	1 (10%)	9 (90%)
Smoking	Yes	17 (57%)	4 (24%)	13 (76%)
	No	13 (43%)	4 (31%)	9 (69%)
Alcohol	Yes	12 (40%)	4 (33%)	8 (67%)
	No	18 (60%)	4 (22%)	14 (78%)
Infection	No	24 (80%)	4 (17%)	20 (83%)
	Yes	6 (20%)	4 (67%)	2 (33%)
Dysplasia	No	22 (73%)	0 (0%)	22 (100%)
	Yes	8 (27%)	8 (100%)	0 (0%)
Malignancy	No	25 (83%)	3 (12%)	22 (88%)
	Yes	5 (17%)	5 (100%)	0 (0%)

## Discussion

The clinical outcomes of surgical excision of oral leukoplakia are complex, as shown in the study results. This study found varying recurrence rates depending on patient characteristics and lesion types, with notable findings such as higher recurrence rates in nonhomogenous lesions, tobacco users, and patients with dysplasia. These findings align with previous studies that emphasized the importance of lesion characteristics and patient risk factors in predicting outcomes after surgical intervention [4,13].

One of the critical observations in this study was the high recurrence rate in nonhomogenous lesions (40%) compared to that in homogenous lesions (0%). This pattern is consistent with the findings of Sundberg et al., who reported that nonhomogenous leukoplakia is associated with a higher risk of malignant transformation and recurrence owing to its varied cellular morphology and propensity for atypical changes [13]. Similarly, Yang et al. noted that nonhomogenous lesions are often indicative of higher dysplastic changes, contributing to poor clinical outcomes after surgery [17]. The existence of erythroplastic regions, ulcerative lesions, and irregular surface texture in nonhomogenous leukoplakia is frequently associated with an increased probability of dysplastic or precancerous alterations.

Another notable outcome was the association between tobacco use and increased recurrence rate. This study showed a recurrence rate of 35% in tobacco users compared to 10% in nonusers, highlighting tobacco as a significant risk factor for poor prognosis. This is in line with previous studies, which concluded that continued use of tobacco after surgery exacerbated the risk of leukoplakia recurrence and increased the likelihood of malignant transformation [16,18]. Tobacco-related carcinogens cause persistent mucosal irritation and genetic alterations, thereby promoting lesion recurrence and malignant transformation.

This study also showed a high recurrence rate for lesions associated with dysplasia (100%). Dysplastic changes are a well-established predictor of poor prognosis, as shown in a previous study, which suggested that oral leukoplakia with dysplasia has a significantly increased risk of recurrence and malignant transformation compared with nondysplastic lesions [12,15]. Therefore, the presence of dysplasia in excised lesions is a crucial factor in determining patient prognosis, and close follow-up is necessary for early detection of recurrence.

Additionally, the study reported a 100% recurrence of malignant lesions. Postexcision malignant transformation or residual malignant cells significantly contribute to poor outcomes. This aligns with the findings of Silverman et al., who emphasized that surgical excision might not always be curative for leukoplakia with malignant changes, necessitating adjunct therapies and rigorous postsurgical monitoring [14,16].

Interestingly, sex differences in recurrence rates were minimal, with 29% recurrence in males compared to 22% in females [12,19]. While this finding was not statistically significant, some studies, such as those by Yang et al., have reported slightly lower transformation rates in female patients, potentially due to lifestyle factors such as reduced tobacco and alcohol use [18].

Clinical significance

This study highlighted that the presence of nonhomogeneous and dysplastic lesions, along with a history of tobacco consumption, notably elevated the likelihood of recurrence following surgical removal of oral leukoplakia. Healthcare professionals should consider these factors when devising postoperative monitoring strategies, customizing follow-up procedures, and providing guidance on modifying risk factors to prevent malignant progression.

Limitations

The major limitation of the present study was the relatively short follow-up period of 18 months, which may not capture long-term recurrence or malignant transformation rates. Furthermore, the present study included only cases in which surgical excision was performed to treat oral leukoplakia, which could have led to selection bias in the study. The results of this study may not be applicable to individuals receiving nonsurgical treatments or those managed through active monitoring strategies. Finally, the small sample size, particularly for subgroups such as sex and lesion type, may have reduced the statistical power, making it difficult to draw definitive conclusions for these subpopulations.

## Conclusions

In conclusion, the clinical outcomes of surgical excision of oral leukoplakia were influenced by lesion characteristics, patient risk factors, and histopathological features, such as dysplasia and malignancy. Nonhomogenous lesions, tobacco use, and dysplastic changes were associated with higher recurrence rates, indicating the need for tailored postsurgical management strategies. Close monitoring and risk factor modification are recommended to optimize long-term outcomes in these high-risk groups.
